# Supplementary material for: Cell-based glycan arrays for probing glycan–glycan binding protein interactions
Source: Nat Commun. 2018 Feb 28;9:880. doi: 10.1038/s41467-018-03245-5 (PMC5830402; doi:10.1038/s41467-018-03245-5)
Supplement: Supplementary file 1 — Supplementary Information [file 41467_2018_3245_MOESM1_ESM.docx]

**Supplementary Information**

**Cell-Based Glycan Arrays for Probing Glycan–Glycan Binding Protein Interactions**

Jennie Grace Briard^1^, Hao Jiang^2^, Kelley W. Moremen^3^, Matthew Scott Macauley*^,1,4^, and Peng Wu*^,1^

^1^Department of Molecular Medicine, The Scripps Research Institute, La Jolla, CA 92037, USA

^2^Key Laboratory of Marine Drugs, Ministry of Education and Qingdao National Laboratory for Marine Science & Technology and Shandong Provincial Key Laboratory of Glycoscience & Glycoengineering, School of Medicine and Pharmacy, Ocean University of China, Qingdao 266003, China

^3^Complex Carbohydrate Research Center and the Department of Biochemistry and Molecular Biology, University of Georgia, Athens, GA 30602, USA

^4^Current address: Department of Chemistry, University of Alberta, Edmonton, AB T6G 2G2, Canada

*Correspondence and requests for materials should be addressed to P.W. (email: pengwu@scripps.edu) or to M.S.M. (email: macauley@ualberta.ca)

**Supplementary Figures**


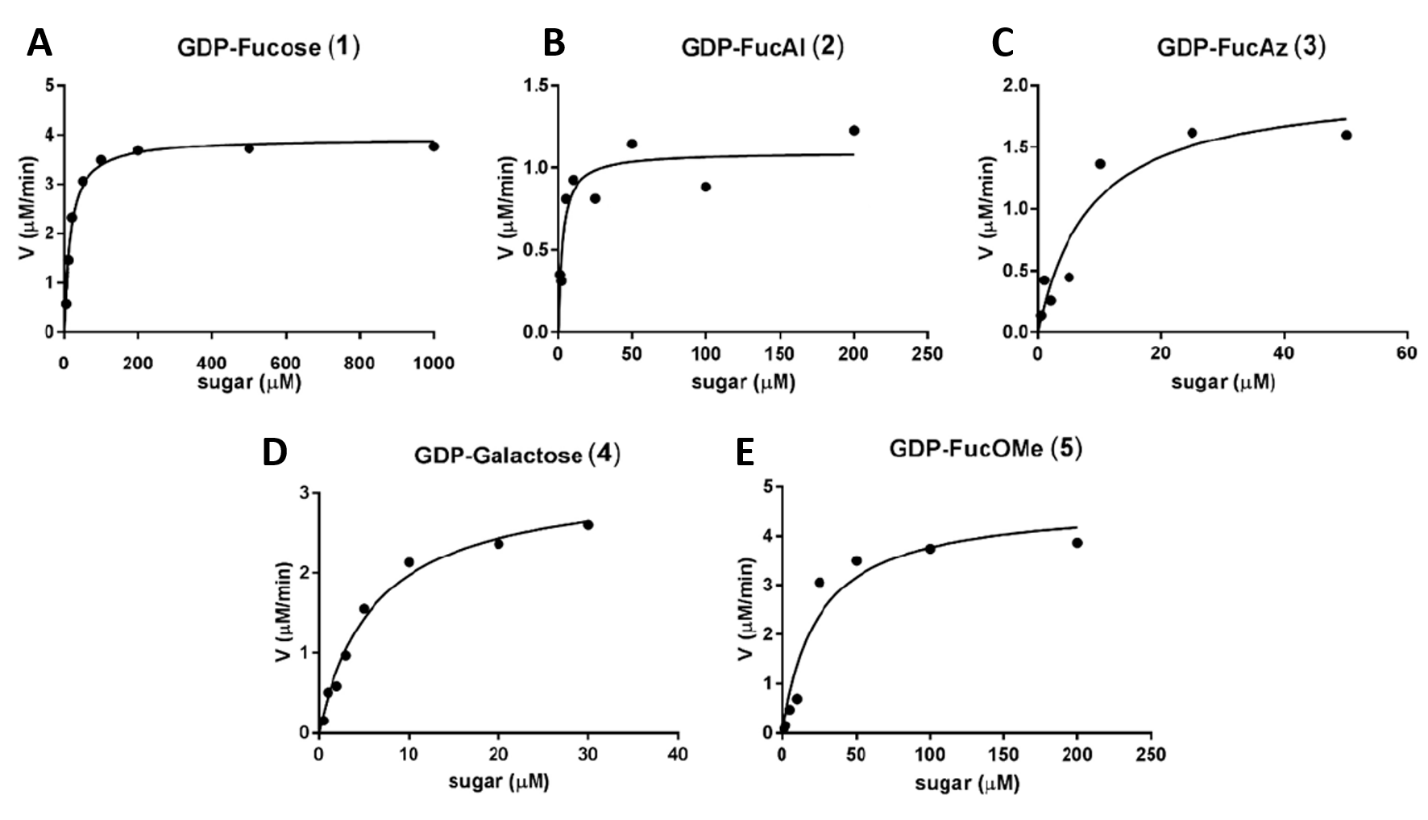


**Supplementary Figure 1. Kinetic analysis of α1-3FT transfer of GDP-fucose analogs** Reaction kinetics for chemoenzymatic transfer to the cell surface of CHO cells using α1-3FT and (A) GDP-fucose (**1**) (K_m_ = 16.3 μM, k_cat_/K_m_ = 13.1 μM^-1^s^-1^), (B) **2** (K_m_ = 2.8 μM, k_cat_/K_m_ = 5.3 μM^-1^s^-1^), (C) **3** (K_m_ = 8.4 μM, k_cat_/K_m_ = 3.2 μM^-1^s^-1^), (D) **4** (K_m_ = 6.2 μM, k_cat_/K_m_ = 10.3 μM^-1^s^-1^), or (E) **5** (K_m_ = 23.9 μM, k_cat_/K_m_ = 2.6 μM^-1^s^-1^).


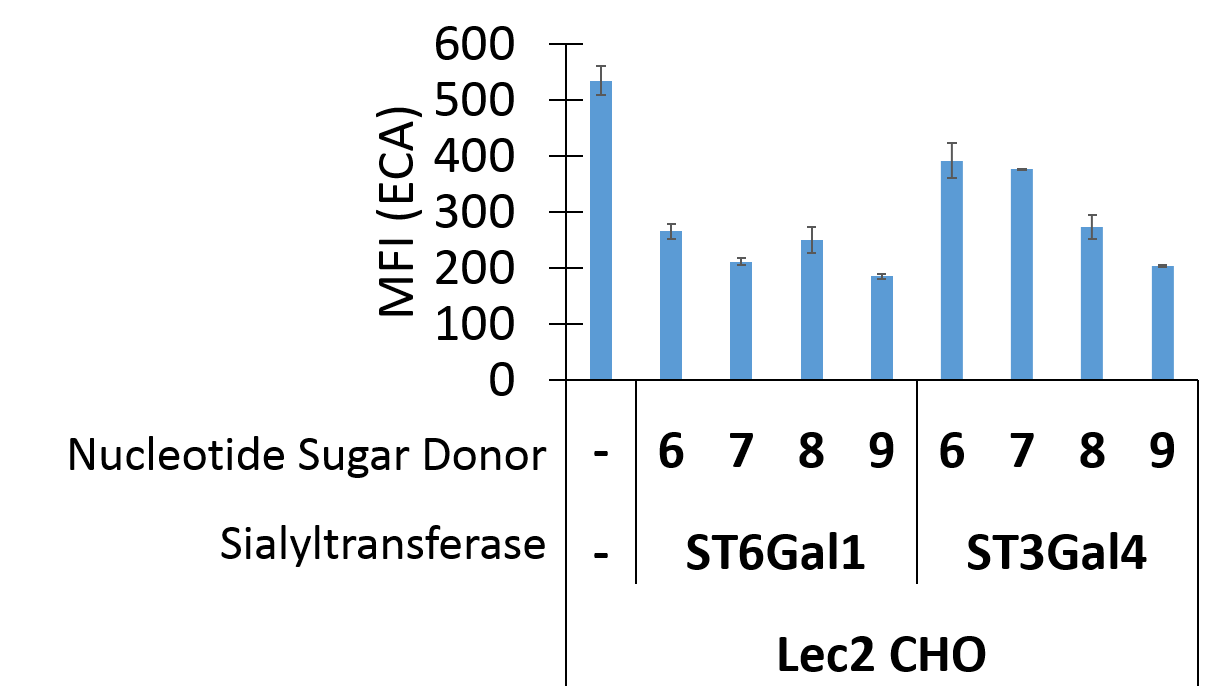


**Supplementary Figure 2. Transfer of unnatural CMP-sialic acid** Lec2 CHO cells were sialylated using CMP-sialic acid (**6**) and analogs (**7**-**8**). ECA interaction was measured after cell-surface sialylation using **6-9** by flow cytometry (n=3, error bars are reported as SEM).

**
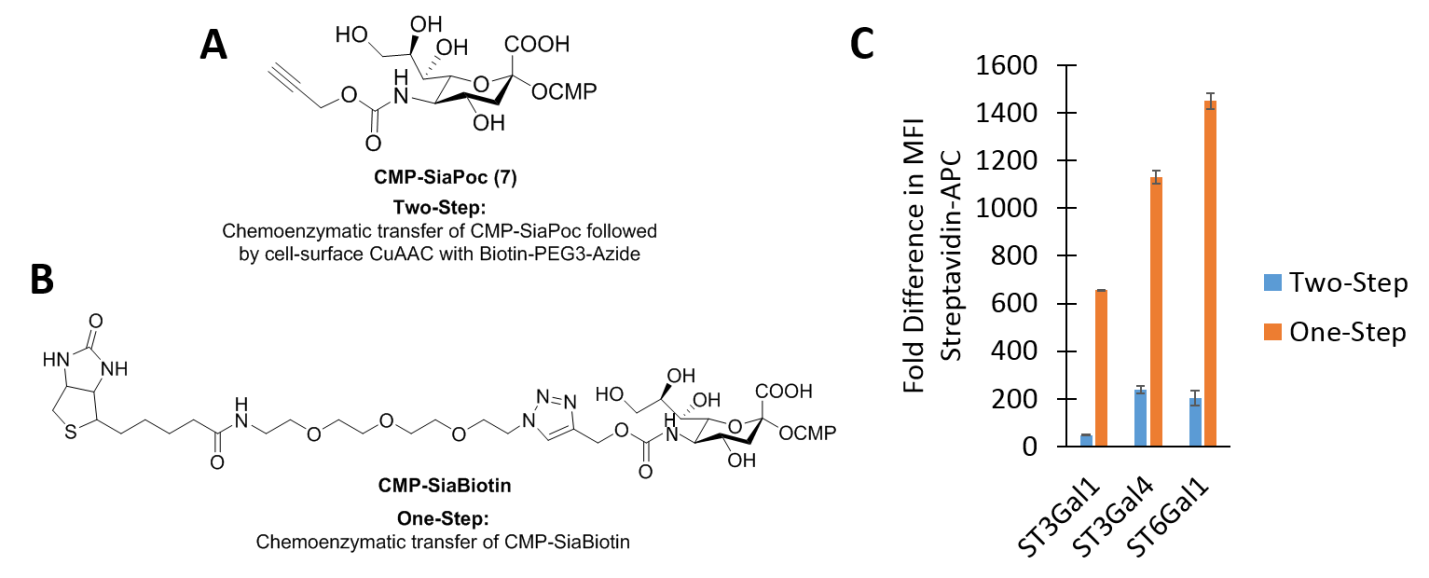
**

**Supplementary Figure 3. Comparison of two-step and one-step transfer** (A) Two-step involves the transfer of CMP-SiaPoc (**7**) by ST3Gal1, ST3Gal4 or ST6Gal1 to Lec2 CHO cells followed by cell-surface CuAAC using Biotin-PEG3-Azide. (B) One-step involves the direct transfer of CMP-SiaBiotin. (C) One-step transfer is more efficient as determined by streptavidin-APC binding by flow cytometry (n=3, error bars are reported as SEM).

**
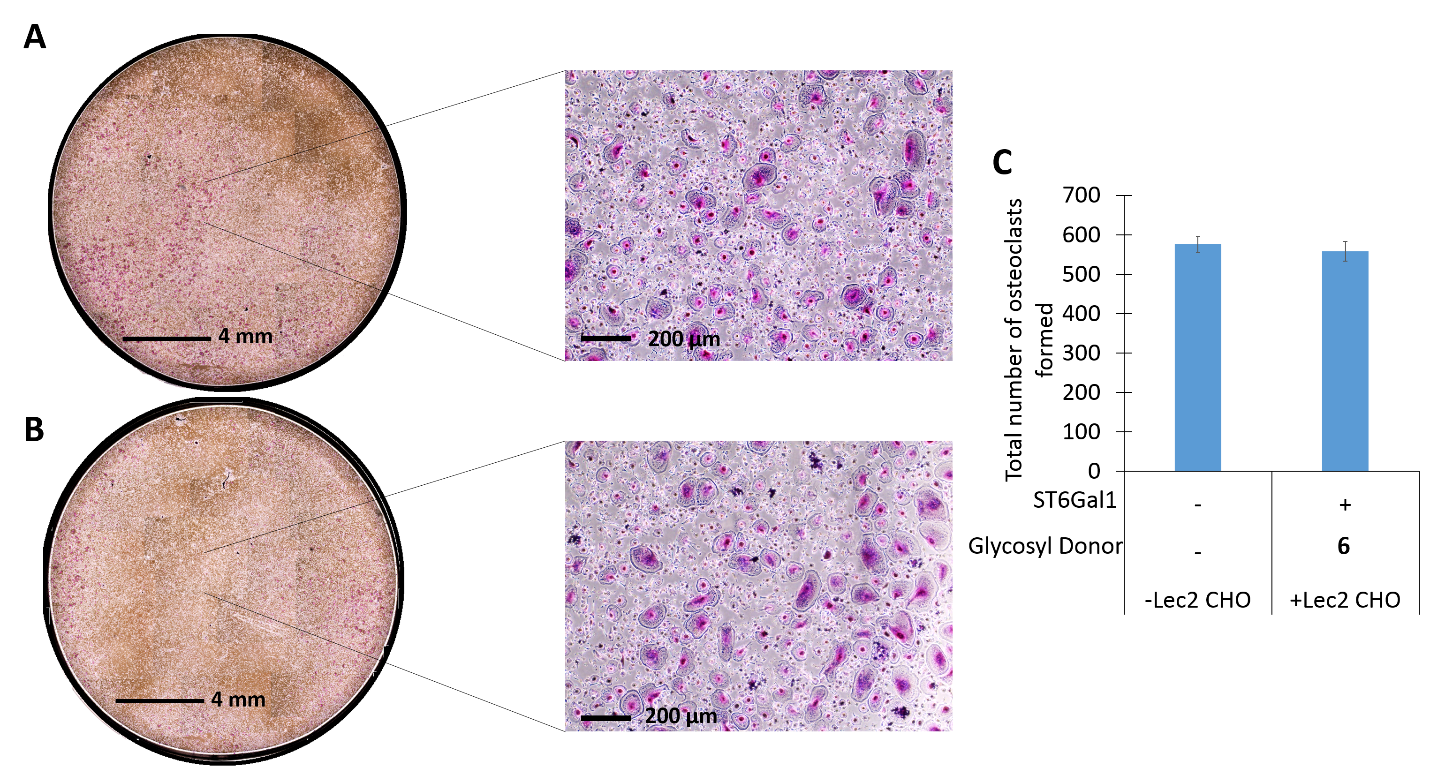
**

**Supplementary Figure 4. Impact of α2-6-6 on osteoclast differentiation** Osteoclast formation measured by TRAP-staining assay after 7 days of differentiation with (A) no addition of Lec2 CHO cells; (B) addition of Lec2 CHO cells labelled with **α2-6-6** on day 5; (C) Total number of osteoclasts formed after 7 days of differentiation (n=3, error bars are reported as SEM).

**Supplementary Figure 5. Chemical structures of FucAl, FucAz, and FucMethoxy**

**Supplementary Figure 6. Synthesis of FucMethoxy (FucOMe)**

**Supplementary Figure 7. Chemoenzymatic synthesis of GDP-fucose analogs**

**Supplementary Figure 8. Chemical structures of ManNPoc, ManNAl, and ManNAz**


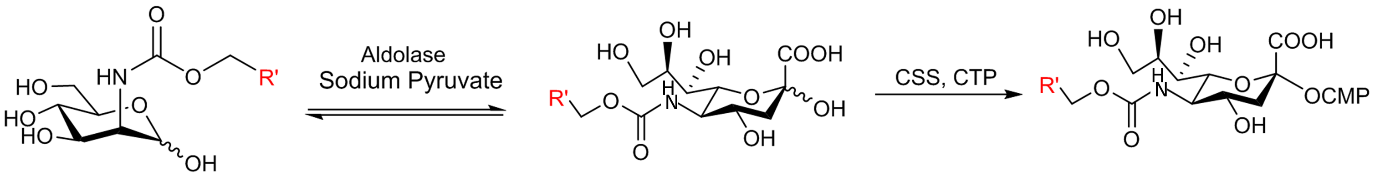


**Supplementary Figure 9. Chemoenzymatic synthesis of CMP-sialic acid analogs**


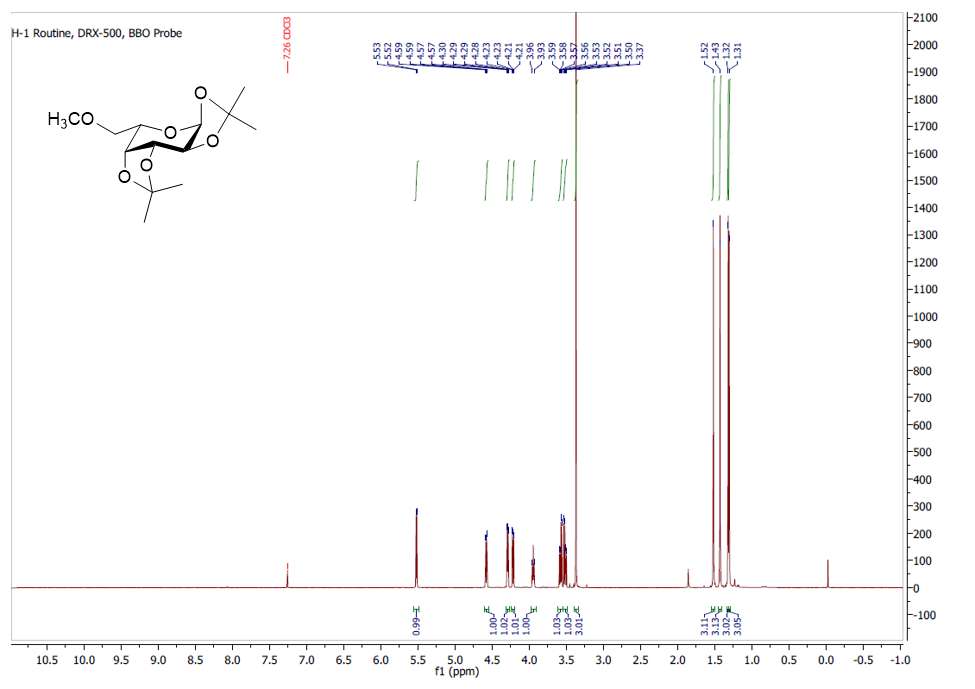


**Supplementary Figure 10. ^1^H NMR spectra of 1,2,3,4-Di-O-Isopropylidene-6-Methoxy-α-l-Galactopyranoside**

**
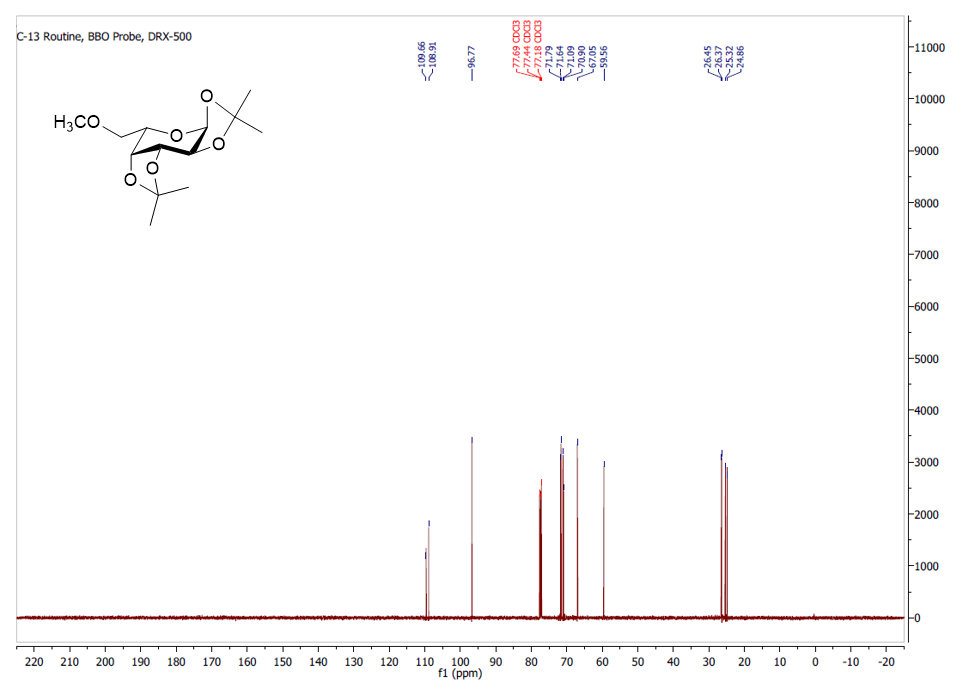
**

**Supplementary Figure 11. ^13^C NMR spectra of 1,2,3,4-Di-O-Isopropylidene-6-Methoxy-α-l-Galactopyranoside**


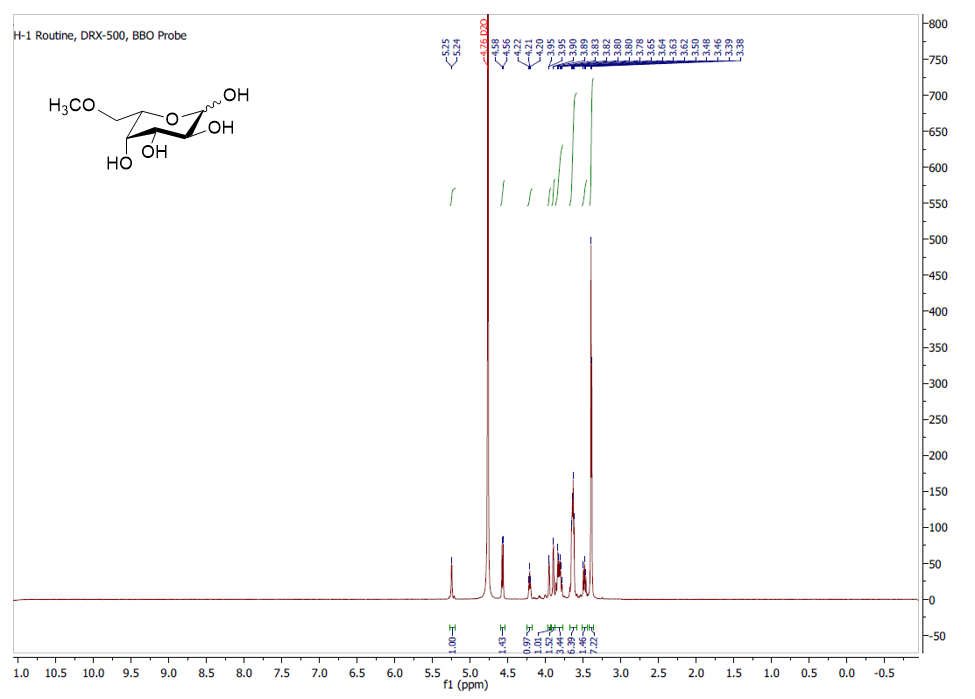


**Supplementary Figure 12. ^1^H NMR spectra of FucOMe**


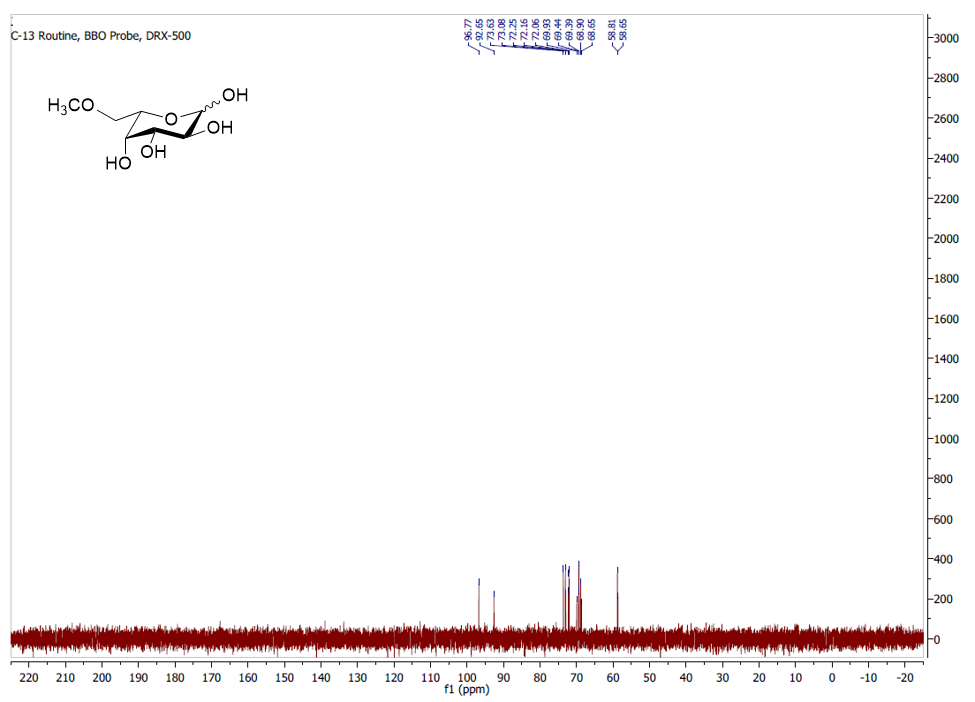


**Supplementary Figure 13. ^13^C NMR spectra of FucOMe**

**Supplementary Tables**

**Supplementary Table 1. Structures of azide library.** Azides 1-40 (A1-A40) used for cell surface copper-I catalyzed azide-alkyne cycloaddition (CuAAC).
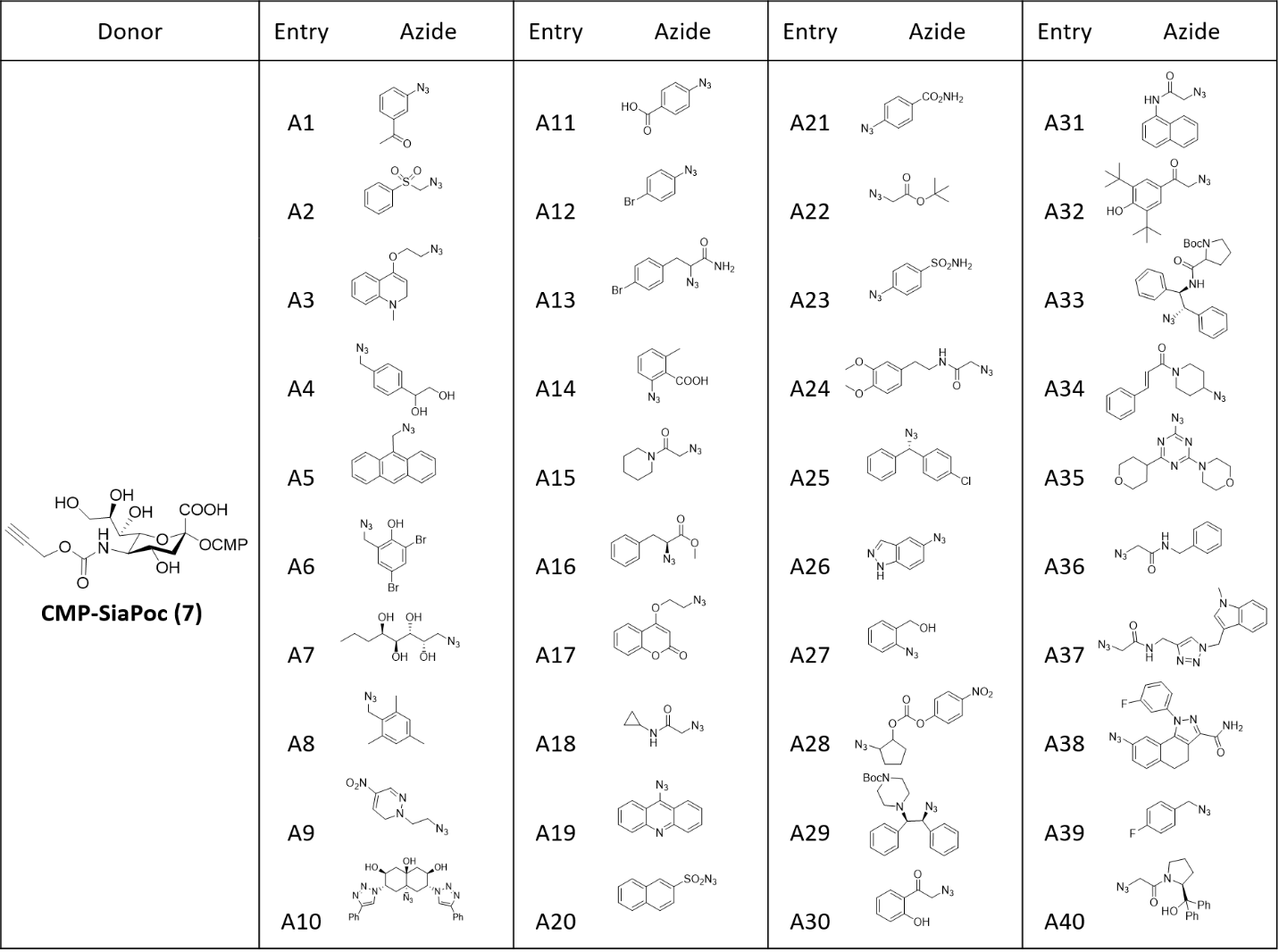


**Supplementary Methods**

**FucAl and FucAz** (Supplementary Fig. 5) were synthesized as previously described.^1, 2^ Briefly, FucAl was synthesized by selective isopropylidene protection followed by IBX oxidation and Seyferth-Gilbert homologation of the C6 hydroxyl group. Subsequent isopropylidene deprotection yielded FucAl. FucAz was synthesized by selective isopropylidene protection followed by treatment with triflic anhydride and C6 triflate displacement using sodium azide. Subsequent isopropylidene deprotection yielded FucAz.

**Synthesis of FucMethoxy** (Supplementary Fig. 6):

**1,2,3,4-Di-O-Isopropylidene-6-Methoxy-α-l-Galactopyranoside** 1,2,3,4-Di-O-isopropylidene-α-l-galactopyranoside^1, 2^ (120 mg, 0.48 mmol) was dissolved in dry DMF (1 mL) and sodium hydride (60% in mineral oil, 29 mg, 0.72 mmol) was added. The reaction mixture was heated to 50 °C and stirred for 30 minutes. After stirring for 30 minutes, the solution was cooled to 0 °C and methyl iodide (102 mg, 0.72 mmol) was added. The reaction mixture was allowed to warm to room temperature overnight. After TLC indicated reaction completion, the reaction was quenched by the addition of saturated ammonium chloride and the product was extracted in ether, washed twice with H_2_O. Purification by flash chromatography (9:1 Hex/EtOAc) yielded pure product, 1,2,3,4-di-O-isopropylidene-6-methoxy-α-l-galactopyranoside (60 mg, 46%). Spectra are found in Supplementary Figures 10 & 11. ^1^H NMR (500 MHz, CDCl_3_): δ 5.53 (d, *J* = 5.0 Hz, 1H), 4.58 (dd, *J* = 7.9, 2.4 Hz, 1H), 4.29 (dd, *J* = 7.5, 2.5 Hz, 1H), 4.22 (dd, *J* = 7.9, 2.0 Hz, 1H), 3.93-3.96 (m, 1H), 3.58 (dd, *J* = 10.0, 5.4 Hz, 1H), 3.52 (dd, *J* = 10.1, 7.0 Hz, 1H), 3.37 (s, 3H), 1.52 (s, 3H), 1.43 (s, 3H), 1.32 (s, 3H), 1.31 (s, 3H). ^13^C NMR (125 MHz, CDCl_3_): δ 109.66, 108.91, 96.77, 71.79, 71.64, 71.09, 70.90, 67.05, 59.56, 26.45, 26.37, 25.32, 24.86.

**6-Methoxy-α-l-Galactopyranoside (FucOMe)** 1,2,3,4-di-O-isopropylidene-6-methoxy-α-l-galactopyranoside (60 mg, 0.22 mmol) was dissolved in 90% TFA in H2O and allowed to stir at room temperature for 2 hr. After TLC indicated reaction completion, the reaction mixture was concentrated in vacuo and product purified by flash chromatography (9:1 DCM/MeOH) to yield FucOMe (39 mg, 91%) as a mixture of α and β anomers. Spectra are found in Supplementary Figures 12 & 13.

**Chemoenzymatic synthesis of GDP-fucose analogs** (Supplementary Fig. 7) GDP-fucose and derivatives were prepared as previously described.^3^ Briefly, L-fucose or its analogs (10 mM), ATP (10 mM), GTP (10 mM), MgSO_4_ (10 mM), inorganic pyrophosphatase (10 units), and FKP (1 unit) were added to Tris-HCl (pH 7.5, 100 mM). The reaction mixture was incubated at 37 °C for overnight with shaking (225 rpm). The reaction was monitored by TLC, using 6:3:2 *i*PrOH:H_2_O:NH_4_OH as the developing solvent and 10% H_2_SO_4_ in EtOH stain. Once complete, the same volume of ice-cold ethanol was added to quench the reaction, the alcoholic mixture was incubated on ice for 30 min. Insoluble material was removed by centrifugation (5,000 x g, 30 min) and the supernatant was concentrated in vacuo to remove volatile ethanol. Crude reaction products were purified by Bio-Gel P2 gel filtration chromatography eluted with NH_4_CO_3_ (50 mM). Only the fractions containing the product were collected and lyophilized.

**ManPoc, ManNAl and ManNAz** (Supplementary Fig. 8) were synthesized as previously described.^2, 4, 5^ Briefly, d-mannosamine hydrochloride was reacted with propargyl chloroformate, 4-pentynoic acid NHS ester or azido acetic acid NHS ester in triethylamine to yield ManPoc, ManNAc and ManNAz derivatives, respectively.

**Chemoenzymatic synthesis of CMP-sialic acid analogs** (Supplementary Fig. 9) CMP-Sialic acid and derivatives were prepared using the one-pot two-enzyme system with *N. meningitidis* CSS and aldolase as previously described.^6^ Briefly, NeuNAc or its analogs (10 mM), CTP (10 mM), sodium pyruvate (20 mM), MgCl_2_ (100 mM), Aldolase (1 mg/mL), and *N. meningitidis* (0.15 mg/mL) were added to Tris-HCl (pH 8.8, 100 mM). The reaction mixture was incubated at 37 °C for 3-4 hours with shaking (225 rpm). The reaction was monitored by TLC, using 6:3:2 *i*PrOH:H_2_O:NH_4_OH as the developing solvent and 10% H_2_SO_4_ in EtOH stain. Once complete, the same volume of ice-cold ethanol was added to quench the reaction, the alcoholic mixture was incubated on ice for 30 min. Insoluble material was removed by centrifugation (5,000 x g, 30 min) and the supernatant was concentrated in vacuo to remove volatile ethanol. Crude reaction products were purified by Bio-Gel P2 gel filtration chromatography eluted with NH_4_CO_3_ (50 mM). Only the fractions containing the product were collected and lyophilized.

**Supplementary References**

1. Yi, W. *et al.* Remodeling bacterial polysaccharides by metabolic pathway engineering. *Proc. Natl. Acad. Sci. U. S. A.* **106**, 4207-4212 (2009).

2. Laughlin, S.T. & Bertozzi, C.R. Metabolic labeling of glycans with azido sugars and subsequent glycan-profiling and visualization via Staudinger ligation. *Nat Protoc* **2**, 2930-2944 (2007).

3. Wang, W. *et al.* Chemoenzymatic synthesis of GDP-L-fucose and the Lewis X glycan derivatives. *Proc. Natl. Acad. Sci. U. S. A.* **106**, 16096-16101 (2009).

4. Yu, H. *et al.* A multifunctional Pasteurella multocida sialyltransferase: a powerful tool for the synthesis of sialoside libraries. *J. Am. Chem. Soc.* **127**, 17618-17619 (2005).

5. Hsu, T.L. *et al.* Alkynyl sugar analogs for the labeling and visualization of glycoconjugates in cells. *Proc. Natl. Acad. Sci. U. S. A.* **104**, 2614-2619 (2007).

6. Yu, H., Yu, H., Karpel, R. & Chen, X. Chemoenzymatic synthesis of CMP-sialic acid derivatives by a one-pot two-enzyme system: comparison of substrate flexibility of three microbial CMP-sialic acid synthetases. *Bioorg. Med. Chem.* **12**, 6427-6435 (2004).
